# Compensatory orthodontic treatment of skeletal Class III malocclusion with anterior crossbite

**DOI:** 10.1590/2176-9451.19.1.113-122.bbo

**Published:** 2014

**Authors:** José Valladares Neto

**Affiliations:** 1 Adjunct Professor, Department of Orthodontics, Federal University of Goiás. Certified by the Brazilian Board of Orthodontics and Facial Orthopedics.

**Keywords:** Crossbite, Tooth extraction, Corrective orthodontics

## Abstract

**Introduction:**

This case report describes the orthodontic treatment of an adult patient with skeletal Class III malocclusion and anterior crossbite. A short cranial base led to difficulties in establishing a cephalometric diagnosis. The patient's main complaint comprised esthetics of his smile and difficulties in mastication.

**Methods:**

The patient did not have the maxillary first premolars and refused orthognathic surgery. Therefore, the treatment chosen was orthodontic camouflage and extraction of mandibular first premolars. For maxillary retraction, the vertical dimension was temporarily increased to avoid obstacles to orthodontic movement.

**Results:**

At the end of the treatment, ideal overjet and overbite were achieved.

**Conclusion:**

Examination eight years after orthodontic treatment revealed adequate clinical stability. This case report was submitted to the Brazilian Board of Orthodontics and Facial Orthopedics (BBO) as part of the requirements to become a BBO diplomate.

## Abstract (pt)

**Introdução:**

o presente relato de caso clínico versa sobre o tratamento ortodôntico em um paciente adulto com má oclusão de Classe III esquelética e mordida cruzada anterior. Dificuldades de diagnóstico cefalométrico foram geradas pela base craniana encurtada. A queixa principal se direcionou à estética do sorriso e a problemas relacionados com a função mastigatória.

**Métodos:**

o tratamento ortodôntico de escolha foi a compensação dentoalveolar por meio da extração de primeiros pré-molares inferiores, uma vez que o paciente apresentava ausência dos primeiros pré-molares superiores e recusou-se à realização da cirurgia ortognática. A retração inferior foi auxiliada pelo levantamento provisório da dimensão vertical da oclusão para que a movimentação ortodôntica ocorresse sem entraves.

**Resultados:**

ao final do tratamento, sobressaliência e sobremordida ideais foram obtidas.

**Conclusão:**

a reavaliação oito anos após o tratamento ortodôntico revelou adequada estabilidade clínica. O presente caso foi apresentado ao Board Brasileiro de Ortodontia e Ortopedia Facial (BBO), como parte dos requisitos para se tornar diplomado pelo BBO.

## INTRODUCTION

A 22-year and 10-month-old male patient arrived for his initial examination in good general health, complaining about his smile, particularly an anterior crossbite and maxillary diastemas, as well as difficulties associated with mastication. His dental history included the extraction of maxillary first premolars at the age of 12, carried out by a clinical dentist due to lack of adequate space for eruption of maxillary canines.

## DIAGNOSIS

**Figure 1:**
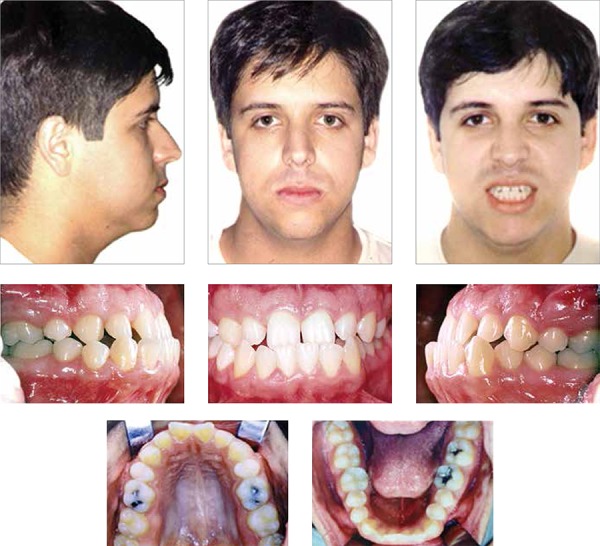
Initial facial and intraoral photographs.

Facial examination revealed balanced characteristics: mesofacial pattern, symmetric features and adequate lip seal. However, a sagittal maxillomandibular deficiency was also noted. The patient had narrow nostrils, slightly ptotic nasal tip, paranasal deficiency, marked grooves at rest and when smiling, a short mentocervical line and an obtuse mentocervical angle, which confirmed the diagnosis. There was also a discrete predominance of maxillary deficiency (Fig 1). The examination of temporomandibular joints revealed bilateral clicking at mandibular opening and closing, maximal mouth opening of 43 mm and an irregular path, but no pain.

**Figure 2:**
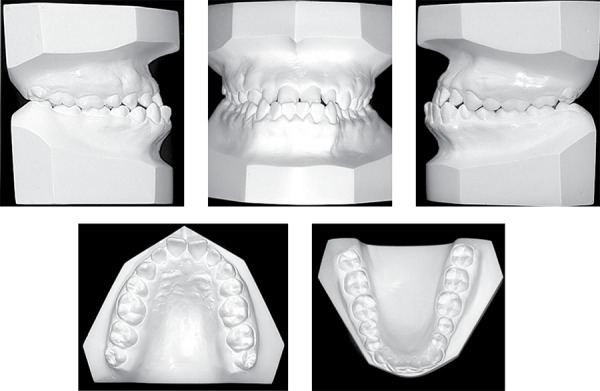
Initial casts.

**Figure 3:**
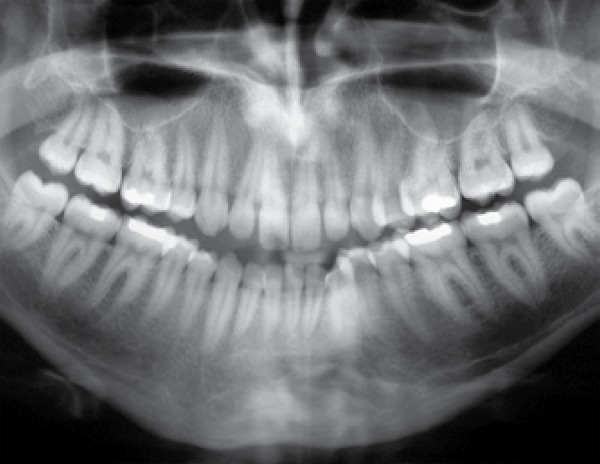
Initial panoramic radiograph.

**Figure 4:**
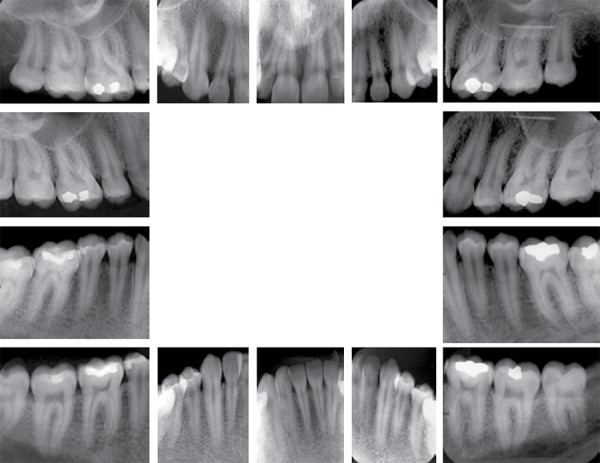
Initial periapical radiographs.

Intraoral clinical examination revealed adequate oral hygiene. Malocclusion was classified as Angle Class I with anterior crossbite, absence of maxillary first premolars, canines in full Class III relationship, anterior mandibular crowding, rotated maxillary central incisors and anterior diastemas (Figs 1 and 2). There were no differences between usual maximal intercuspation and centric relation. Radiographs showed that the patient had good dental and periodontal health and no endodontic problem or bone loss (Figs 3 and 4).

**Figure 5:**
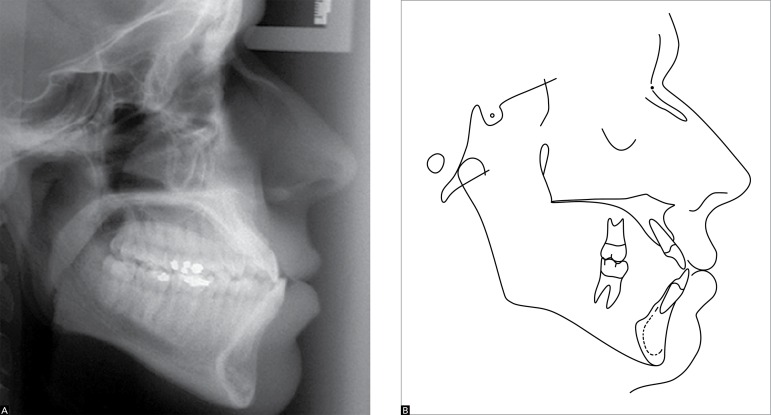
A) Initial cephalometric profile radiograph and B) cephalometric tracing.

**Figure 6:**
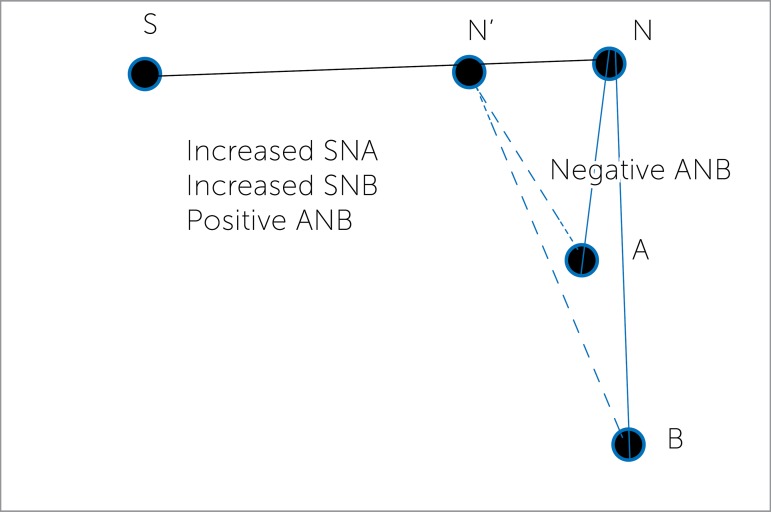
Diagram illustrating Class III skeletal relationship with short (N') and normal (N) anterior cranial bases.

Cephalometry revealed that the maxillomandibular relationship was apparently normal (ANB = 2°) and that a few angles were slightly greater than normal (Conv. = 5.5°; SNA = 86°; SNB = 84°) (Fig 5). However, the ANB angle is known to be markedly affected by geometrical factors.^1^ When the cranial base is short, maxillomandibular discrepancies cannot be evaluated on the sagittal plane using the ANB angle (Fig 6). Other cephalometric parameters (Wits = -8 mm; S-N = 71.5 mm) and particularly facial analysis should be used to elucidate this confounding factor.^2,3^

When evaluated by cephalometry and having the cranial base as reference, maxillary and mandibular incisors showed buccal inclination and marked protrusion (1-NA = 25°, 1-NA = 7 mm) in mandibular teeth (1-NB = 34°, 1-NB = 11 mm). In contrast, the inclination of mandibular incisors in relation to the mandibular plane was good and met the Brazilian standards (IMPA = 94°).^2^

## TREATMENT PLAN

The first treatment plan presented to the patient was the orthodontic combined with orthognathic surgery, which the patient promptly refused. For this reason, an alternative plan was suggested. It included orthodontic camouflage with orthodontic appliances in both arches and extraction of mandibular first premolars. The patient had undergone extraction of maxillary first premolars and, therefore, our aim was to achieve normal molar and canine occlusion. Mandibular extractions followed by retraction of anterior teeth should be supported by adequate anchorage control.

The dentist and the patient agreed on the following objectives for the treatment selected: preservation of maxillary and mandibular bones position; alignment and reduction in maxillary diastemas; alignment of mandibular teeth; normal occlusion, correction of negative overjet and functional occlusion; esthetic improvement after lower lip retraction; and achievement of a pleasant smile.

Treatment plan was divided into the following phases: modified Nance lingual arch (away from mandibular incisors); fixed orthodontic appliances in both arches using the straight-wire system and 0.022 x 0.028-in slots; extraction of mandibular first premolars; tooth leveling and alignment with 0.012-in, 0.014-in and 0.016-in nickel-titanium wires and 0.018-in, 0.020-in and 0.017 x 0.025-in stainless steel wires; retraction of mandibular anterior teeth using sliding mechanics and 0.019 x 0.025-in stainless steel wire; removal of lingual arch; orthodontic treatment finishing; retention.

## TREATMENT PROGRESSION

Treatment progression was in accordance with the plan. Mandibular second molars were included in initial leveling to aggregate an anchorage unit for the retraction of incisors. Maxillary second molars were bonded and included in leveling during orthodontic finishing.

**Figure 7:**
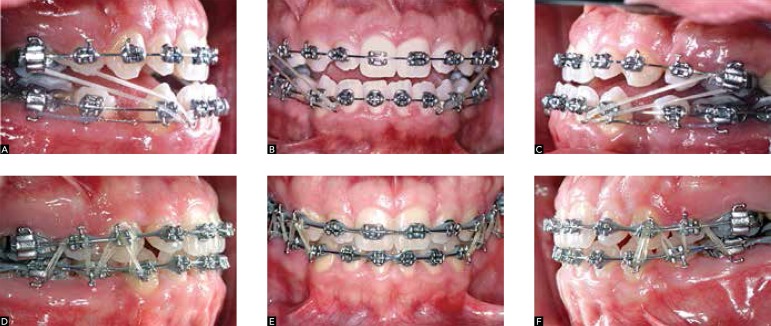
Increased vertical dimension during retraction of mandibular anterior teeth (A, B, C) and treatment completion phase (D, E, F).

**Figure 8:**
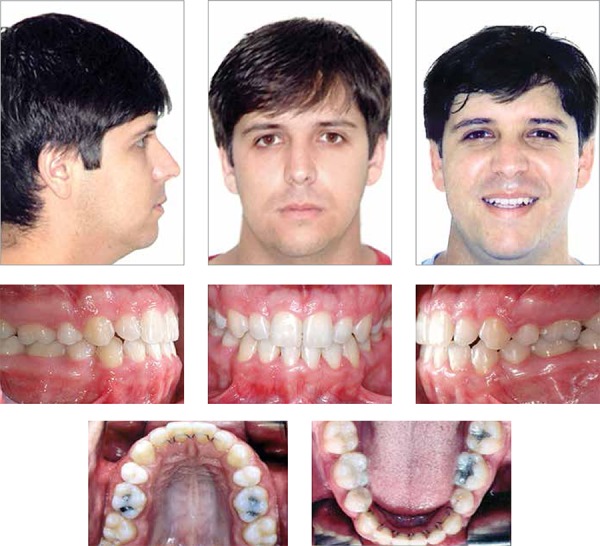
Final facial and intraoral photographs.

**Figure 9:**
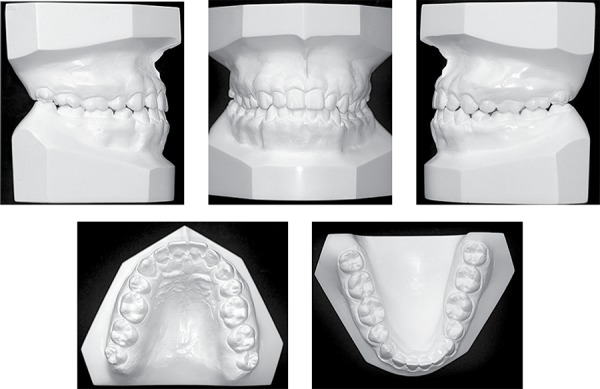
Final casts.

The vertical dimension had to be temporarily increased with glass-ionomer cement built-up on posterior teeth. This procedure was used for retraction of mandibular anterior teeth because anterior crossbite and marked overjet were obstacles to movement (Figs 7A, B and C). Spaces were closed by means of sliding mechanics (0.019 x 0.025-in wire) and hooks were soldered between canines and lateral incisors. Class III intermaxillary elastics (1/4-in, medium force) were used to control anchorage together with the lingual arch which was removed after retraction of anterior teeth and closing of extraction spaces. No skeletal anchorage was used. Treatment was completed with 0.018-in archwires, elastic chains in both arches to retain interproximal contacts, and Class II intermaxillary elastics (5/6-in, medium force) to retain the movement achieved (Figs 7D, E and F). After orthodontic completion, intercuspation was good, and canine and molar occlusion relationships, as well as overjet, were normal (Fig 8, 9). Maxillary (2 x 2) and mandibular (4 x 4) V-looped braided bonded lingual archwires were placed for retention.

## RESULTS

**Figure 10:**
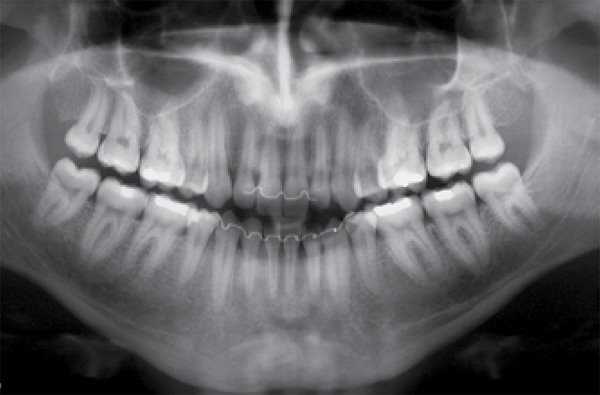
Final panoramic radiograph.

**Figure 11:**
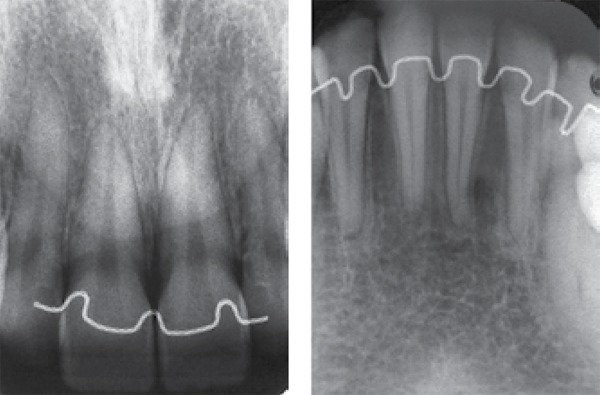
Final periapical radiographs of maxillary and mandibular incisors.

The final radiograph showed that root parallelism was good after space closure and that root size was preserved (Figs 10 and 11).

**Figure 12:**
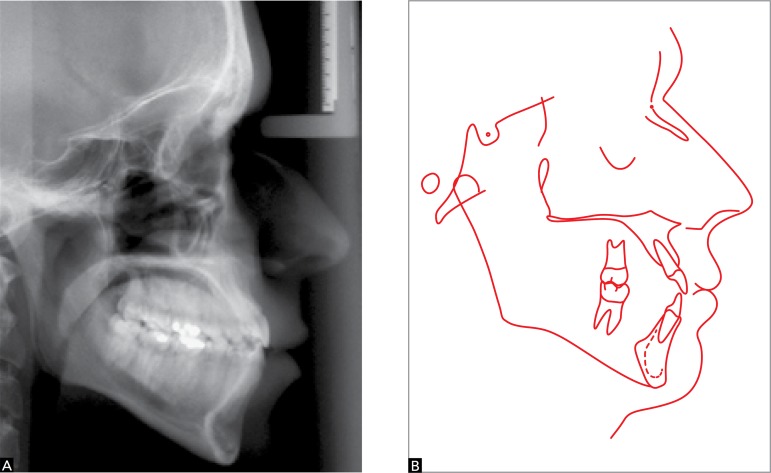
A) Final cephalometric profile radiograph and B) cephalometric tracing.

In the maxillary arch, diastemas were reduced, molars were slightly extruded, intercanine distance (35.5 mm) was preserved and intermolar distance was shortened (from 43.5 mm to 42.0 mm). A marked cephalometric effect was found in the mandibular arch with anterior retraction, intrusion and mesial movement of mandibular molars (Fig 12 and Table 1). However, intercanine (21.5 mm) and intermolar (33.0 mm) distances did not change.

**Table 1:** Initial (A) and final (B) cephalometric values.

	Measures		Normal	A	B	A/B diff.
**Skeletal pattern**	SNA	(Steiner)	82°	86°	85°	1°
	SNB	(Steiner)	80°	84°	83°	1°
	ANB	(Steiner)	2°	2°	2,5°	-0,5°
	Facial angle	(Downs)	0°	5,5°	6,5°	-1,0°
	Y axis	(Downs)	59°	57°	57°	0°
	Facial angle	(Downs)	87°	93°	92°	1°
	SN-GoGn	(Steiner)	32°	34°	34°	0°
	FMA	(Tweed)	25°	26°	23°	3°
**Dental pattern**	IMPA	(Tweed)	90°	94°	78°	16°
	1.NA	(Steiner)	22°	26°	25°	1°
	1-NA	(Steiner)	4mm	7mm	6mm	1mm
	1.NB	(Steiner)	25°	34°	17°	17°
	1-NB	(Steiner)	4mm	11mm	4mm	7mm
	1.1 – Interincisal angle	(Downs)	130°	115°	135°	-20°
	1-APo	(Ricketts)	1mm	10mm	5mm	5mm
**Profile**	Upper lip – S line	(Steiner)	0mm	0mm	0mm	0mm
	Lower lip – S line	(Steiner)	0mm	3mm	0,5mm	2,5mm

**Figure 13:**
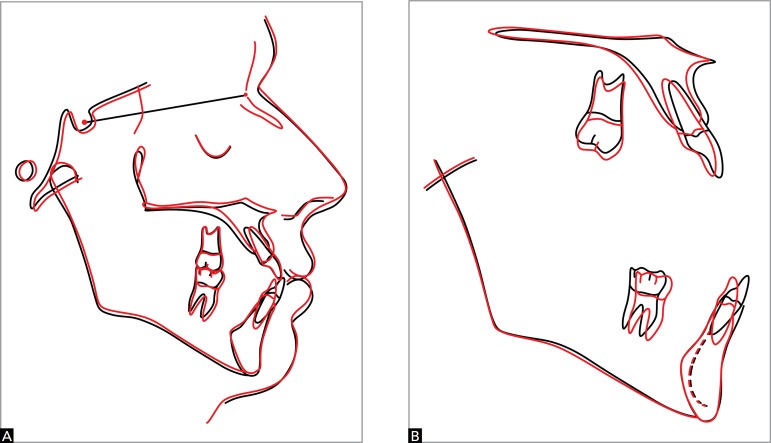
A) Total and B) partial superimpositions of initial (black) and final (red) cephalometric tracings.

**Figure 14:**
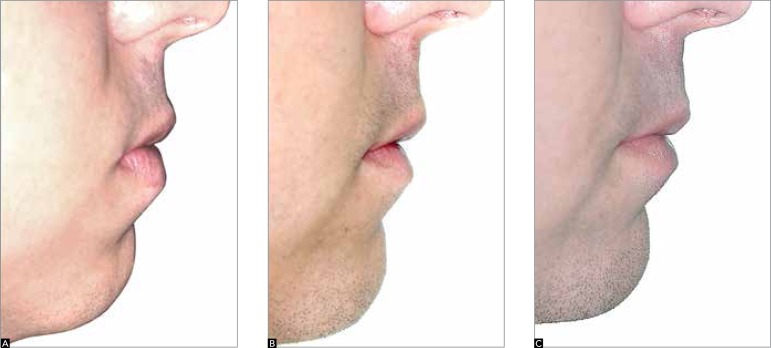
Comparison of facial profile close-up: A) initial, B) final and C) control eight years later.

There were no significant changes in the position of the maxilla or the mandible (Fig 13). Facial esthetics improved due to less marked lower lip protrusion, confirmed by reduction of 2.5 mm in the cephalometric variable that describes the lower lip (S line) (Fig 14 and Table 1).

**Figure 15:**
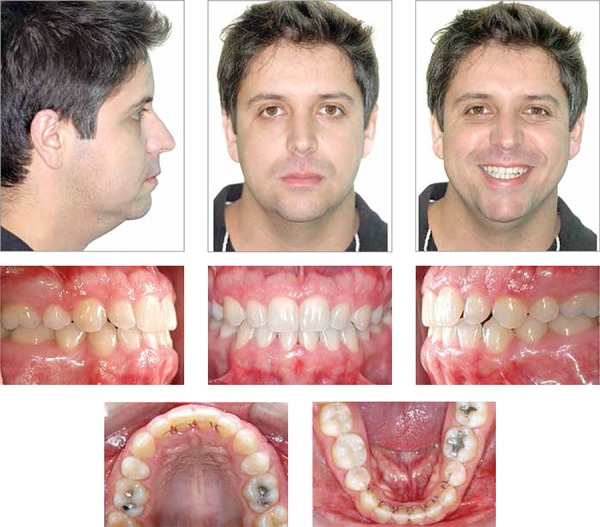
Facial and intraoral control photographs eight years after treatment completion.

The relationship between the maxilla and the mandible showed good intercuspation and coordination, although sagittal skeletal discrepancy was camouflaged by dental compensation. Overjet and overbite were fully corrected, and the criteria for ideal functional occlusion were met. The positive results, confirmed by clinical stability eight years after treatment completion, were favored by the lack of remaining facial growth, the use of fixed retention and patient's satisfactory occlusal relationship (Fig 15).

## FINAL CONSIDERATIONS

Cranial base abnormalities strongly affect the interpretation of cephalometric variables in this region, particularly SNA, SNB, ANB and convexity angle. Other cephalometric parameters, correction factors and, above all, facial analysis findings contributed to making the diagnosis and developing a treatment plan. In adults, Class III skeletal patterns may often be treated with either orthodontic camouflage or orthognathic surgery.^4,5^ In the case reported here, the treatment chosen was orthodontic camouflage with extraction of mandibular first premolars. Treatment results were satisfactory, and the occlusal objectives were achieved. The final harmonious smile pleased the patient and improved his self-esteem and quality of life.
